# Supplementary material for: Exploration of crystal chemical space using text-guided generative artificial intelligence
Source: Nat Commun. 2025 May 12;16:4379. doi: 10.1038/s41467-025-59636-y (PMC12069578; doi:10.1038/s41467-025-59636-y)
Supplement: Supplementary file 1 — Supplementary Information [file 41467_2025_59636_MOESM1_ESM.pdf]

Table of Contents

Supplementary Note S1. Model evaluation on structural and guiding metrics with random split.

Supplementary Note S2. Summary of workflow to navigate chemical systems.

Supplementary Note S3. Benchmarking Chemeleon with other methods.

Supplementary Note S4. Chemeleon trained with mineral types.

Supplementary Figure S1. Results of contrastive learning when textual description is formatted text form.

Supplementary Figure S2. Results of contrastive learning when textual description is general text form.

Supplementary Figure S3. Examples of general text type for textual description.

Supplementary Figure S4. A t-SNE plot of structural embedding for train, test, sampled structures.

Supplementary Figure S5. Ternary phase diagrams of Li-P-S-Cl with chemical filter.

Supplementary Figure S6. A summary of workflows for navigating chemical system.

Supplementary Figure S7. A t-SNE plot of structural embeddings for TiO_2 polymorphs system.

Supplementary Figure S8. Distributions of MACE-MP energies for generated structures by Chemeleon.

Supplementary Figure S9. Novel metastable TiO_2 polymorphs.

Supplementary Figure S10. Novel generated structures in Ti-Zn-O system.

Supplementary Figure S11. Novel generated metastable quaternary structures in Li-P-S-Cl system.

Supplementary Figure S12. DFT phonon band structures in Li-P-S-Cl system.

Supplementary Figure S13. Novel generated stable structures in Li-P-S-Cl system.

Supplementary Figure S14. Known materials for binary systems in Li-P-S-Cl.

Supplementary Figure S15. Details of dataset for Chemeleon.

Supplementary Figure S16. Number of atoms in MP-40 test set with different splits.

Supplementary Figure S17. Benchmarking Chemeleon with other methods.

Supplementary Figure S18. Energy distribution in generated crystal structures in benchmarking results.

Supplementary Figure S19. Chemeleon trained with mineral names.

Supplementary Table S1. Model evaluation on MP-20 dataset.

Supplementary Table S2. Evaluation on text-guided metrics.

Supplementary Table S3. Model evaluation on structural metrics with random split.

Supplementary Note S1. Model evaluation on structural and guiding metrics with random split.

To ensure a robust evaluation across different dataset splitting methods, we also assessed the performance of Chemeleon trained with a random split on the MP-40 dataset, as shown in Supplementary Table S3. This random split exhibit improved performance in structure matching, metastability, composition matching, and crystal system matching compared to the time-based split. This improvement can be attributed to the increased complexity of newer entries added to the Materials Project after August 2, 2018. As shown in Supplementary Figure S16, the distribution of the number of atoms per unit cell varies significantly between the two splitting methods. While the test set in the time-based split predominantly consists of structures with more atoms, the test set in the random split yields a more balanced distribution of atom counts.

Supplementary Note S2. Summary of workflow to navigate chemical systems.

To systematically explore complex chemical systems, we have developed a workflow integrating multiple computational tools. This workflow is designed to filter, generate, screen, thereby facilitating the design and discovery of novel materials.

The workflow begins by narrowing down the chemical space using chemical filters. SMACT applies a series of chemical rules (charge balancing and electronegativity balancing) and filters to identify allowed compositions, effectively reducing the vast chemical space to a manageable set of candidates for further investigation. This step excludes compositions that do not meet specific chemical criteria, thereby focusing efforts on those with a higher likelihood of stability. Taking Zn-Ti-O system as an example, we start with a total of 728 possible compositions, determined by a maximum stoichiometric coefficient of 8, which is calculated as 9^3-1 (excluding the case where all stoichiometric coefficients are zero). Using the *smact_validity* function, only 179 unique, reduced compositions are filtered where they follow the chemical rules.

Following the identification of viable compositions, Chemeleon generates structures by taking these compositions as textual descriptions. The model requires two inputs which are the textual input of the composition and the number of atoms in the unit cell. To ensure a comprehensive exploration of structural possibilities, we consider various multiplicities (Z numbers) for each composition up to Z=4. For instance, a composition such as "Zn3 Ti2 O1" with a Z=2 corresponds to a unit cell comprising 12 atoms. Then, it samples 100 structures for each combination of composition and Z. Duplicate structures are then systematically removed using the `StructureMatcher` function from Pymatgen, resulting in a final set of 16,694 unique structures for the Zn-Ti-O system.

The next stage involves a preliminary screening of these generated structures using machine learning force field, MACE-MP which was pre-trained with 1.5M relaxation trajectories from the Materials Project. These structures undergo geometry optimization with MACE-MP using an iterative algorithm that involves both cell relaxation and internal relaxation while fixing the cell. This optimisation process, with a force convergence threshold of 0.01 eV/Å, yields 16,003 converged structures. To identify metastable structures (defined as those with an energy above the convex hull of less than 0.15 eV/atom

in this work), we applied a tighter energy criterion of less than 0.10 eV/atom, resulting in the identification of 5,014 candidate structures. Given that MACE-MP predicts uncorrected energies, we used uncorrected energies from the Materials Project for the calculation of energy above the convex hull. Due to the substantial number of structures, we selected the 63 structures with the most stable MACE-MP energies for each unique composition to undergo DFT calculations using Atomate2's *MPGGADoubleRelaxStaticMaker*.

The calculated DFT values were subsequently corrected using Pymatgen's *MaterialsProject2020Compatibility* class to ensure accurate phase diagram plotting, as depicted in Figure 4(a). This workflow was applied consistently across different chemical systems to systematically explore and navigate their potential configurations.

Supplementary Note S3. Benchmarking Chemeleon with other methods.

Beyond evaluating Chemeleon using the MP-40 test set, we benchmarked its performance against other structure generation methods to assess for practical use cases. This benchmarking study focuses on three compositions within the Zn-Ti-O system: ZnO, TiO_2, and $TiZnO_3$, considering different integer variations of $TiZnO_3$ such as $Ti_2Zn_2O_6$ and $Ti_4Zn_4O_{12}$. We compared Chemeleon with three other methods: DiffCSP, a diffusion-based crystal structure prediction (CSP) model; CrystaLLM, a large language model (LLM)-based approach; and an element substitution method based on machine learning.

For this study, we sampled 100 structures for each composition using each method and evaluated their performance based on composition match, validity, uniqueness, and thermodynamic stability, as shown in Supplementary Figure S17 and S18. The thermodynamic stability of the generated structures was assessed using the MACE-MP-0 machine-learning force field, which we utilized throughout our study.

In terms of composition matching, DiffCSP and element substitution methods are developed for CSP task, without additional optimisation of composition prediction. They inherently achieve a perfect composition matching score of 1.0. For Chemeleon, we used compositon-based model of which inputs are string format of composition. While it shows almost perfect composition accuracy for relatively simple compositions such as ZnO and TiO_2, the composition matching performance declines as stoichiometry increases and the unit cell size becomes larger. For instance, In the case of $Ti_4Zn_4O_{12}$, some generated structures deviated to $Ti_3Zn_5O_{12}$ or $Ti_4Zn_3O_{13}$. In contrast, CrystaLLM exhibited strong composition generation capabilities.

The validity of generated structures was evaluated based on structural abnormalities, including atomic overlap or excessively large unit cell parameters (greater than 60 Å). All methods predominantly generated valid structures, with a few invalid structures observed in CrystaLLM for $Ti_2Zn_2O_6$.

To evaluate uniqueness, we analysed the proportion of unique structures using `StructureMatcher` class from pymatgen. The element substitution method exhibits the highest uniqueness compared to other generative AI methods. This is due to the nature of substitution methods, which generate structures from diverse structural prototypes, leading to a broader range of unique crystal structures.

Supplementary Figure S18 shows that distribution of the energy per atom based on MACE-MP-0 model. The predicted structures by the substitution method are very broad, indicating that most of the generated structures have higher energy and may be less stable. CrystaLLM exhibits an energy distribution peak in a relatively unstable region compared to DiffCSP and Chemeleon. Furthermore, the energy distribution of CrystaLLM-generated structures is more sparsely spread out, rather than concentrated at lower energy values.

Overall, DiffCSP, which is optimized exclusively for CSP rather than composition prediction, generates structures with the lowest energy distribution peak, indicating superior performance of generating stable structures. Chemeleon, which is optimized to generate crystal structures from textual input, produces structures with an energy distribution comparable to DiffCSP, while also demonstrating flexibility in handling composition variations.”

Supplementary Note S4. Chemeleon trained with mineral types.

As a proof-of-concept for the effectiveness of text-guided generation, we trained the Chemeleon model using human-recognized mineral name conventions (e.g., perovskite, Heusler, ilmenite). We collected mineral names via Robocrystallography based on the AFLOW prototype database for the MP-40 dataset, including only those with at least 10 occurrences. This resulted in a total of 6,537 structures. The most common mineral names included "(Cubic) Perovskite" and "Orthorhombic Perovskite," as well as other well-known names such as Spinel and Rocksalt (see Supplementary Figure S19(a)). We randomly split this dataset into 6,000 training structures and 537 test structures.

For evaluation, we sampled 20 structures from the 537 test structures using their mineral name as text inputs and applied the same structure-matching metrics used previously. This resulted in a 0.86 mineral-matching rate.

To further showcase perovskite generation, we sampled 100 structures covering all unique atomic numbers in the training set with "Perovskite" as text inputs. From these, we identified 112 newly generated perovskite structures that did not overlap with the original training or test sets. Among these, three representative examples are shown in Supplementary Figure S19(b). These range from the simple ABX_3-type perovskite (e.g., $YFeO_3$, formed via element substitution) to more complex quaternary and quinary perovskites. This result demonstrates the model's capability to generate novel materials based on intuitive, human-recognized text inputs.

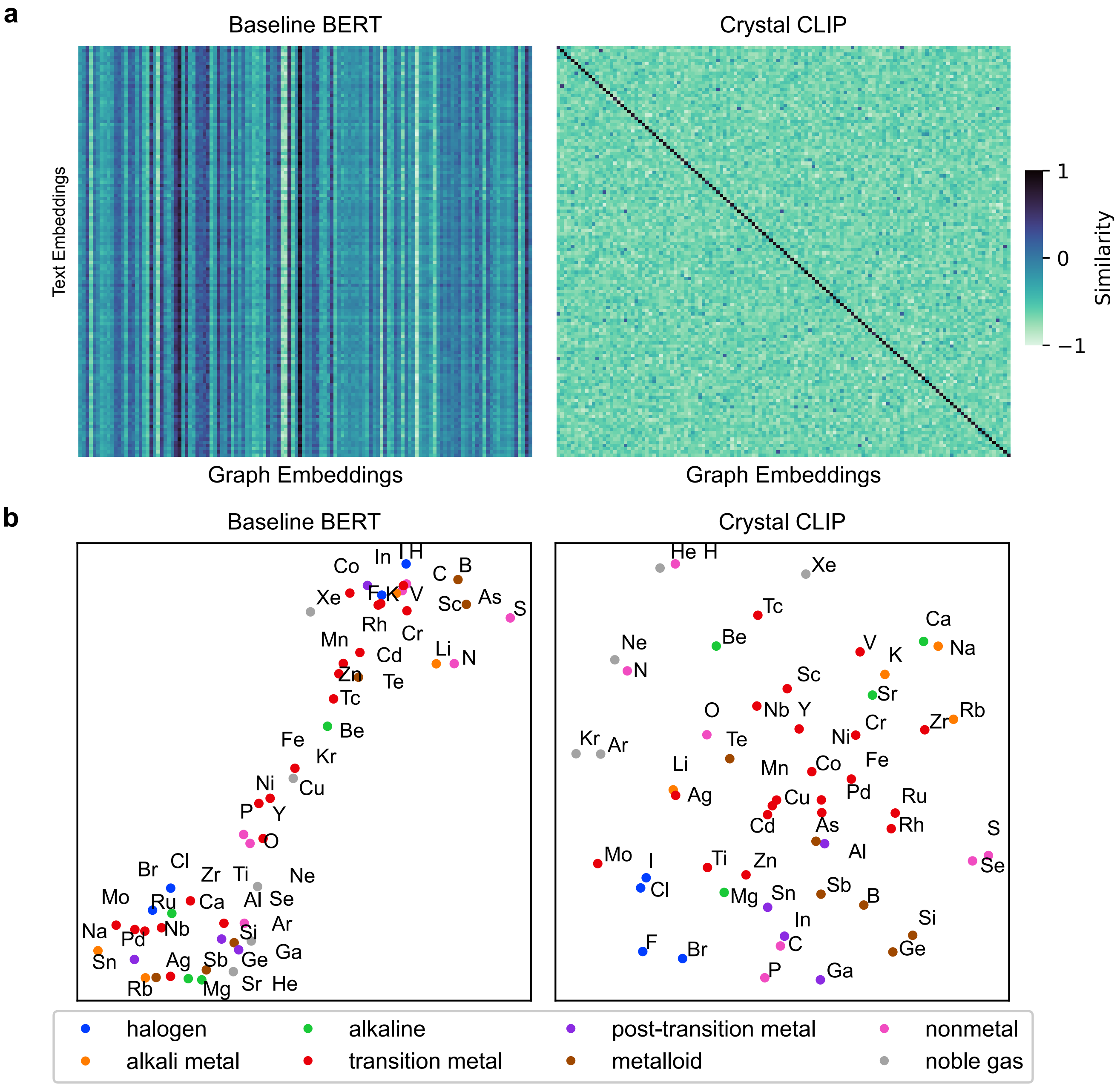

Supplementary Figure S1. Results of contrastive learning when the textual description is in formatted text form. (a) Heatmaps of cosine similarity between text embeddings from text encoders and graph embeddings from GNNs. The Baseline BERT model refers to MatTPUSciBERT. Values are plotted for 128 randomly sampled unit cells, forming a 128×128 matrix. Diagonal elements represent positive pairs, while off-diagonal elements represent negative pairs. (b) A t-SNE visualization of element embeddings generated by the text encoders, using element symbols as the textual input.

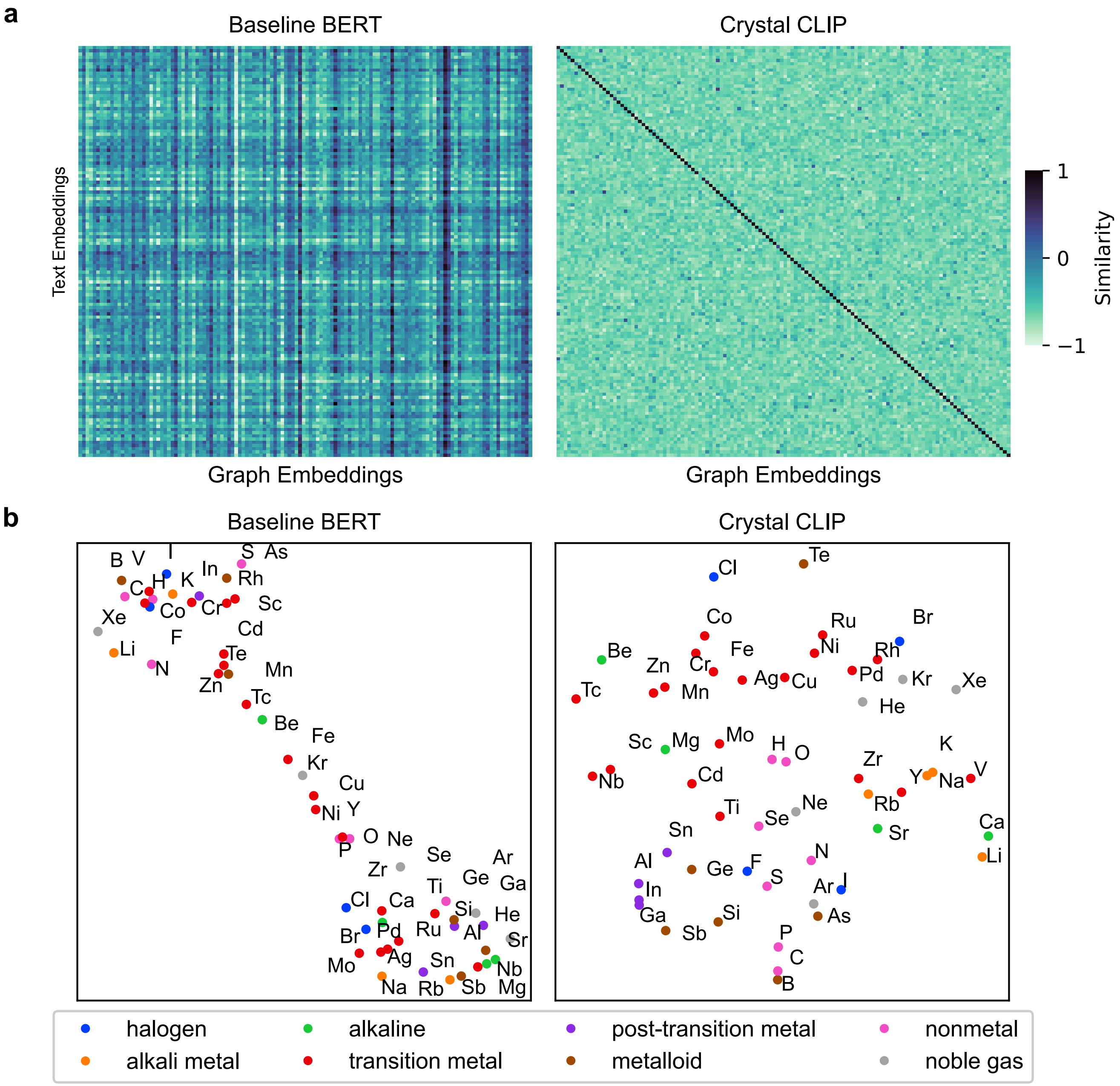

Supplementary Figure S2. Results of contrastive learning when the textual description is in general text form. (a) Heatmaps of cosine similarity between text embeddings from text encoders and graph embeddings from GNNs. The Baseline BERT model refers to MatSciTPUBERT. Values are plotted for 128 randomly sampled unit cells, forming a 128×128 matrix. Diagonal elements represent positive pairs, while off-diagonal elements represent negative pairs. (b) A t-SNE visualization of element embeddings generated by the text encoders, using element symbols as the textual input.

Text Prompt

Provide five concise captions for "{reduced_formula}, {crystal_system}"

Here are some examples for other crystal systems:
1. Orthorhombic crystal structure of ZnMnO4
2. Crystal structure of LiO2 in orthorhombic symmetry
3. Cubic symmetry in SiC crystal structure

Please provide five captions for the crystal structure of {reduced_formula} in {crystal_system} symmetry.

Examples

TbGePd2 crystal structure exhibiting orthorhombic symmetry

Tetragonal Fe3B crystal lattice arrangement

Monoclinic crystal structure of Tl2Ag3(PO4)3

Hexagonal crystal lattice of Mo3O10 compound

Structural analysis of SF4 in orthorhombic symmetry

TbFe5P3 crystal lattice displaying orthorhombic symmetry

Distinctive monoclinic symmetry of MnH4(CO3)2 crystal

Monoclinic symmetry in Li2CuB4(PbO5)2 crystal structure

CsH5N2 crystal structure displaying orthorhombic symmetry characteristics

Monoclinic Tb2CoGe2 crystal lattice

Rb3IO5 crystal system exhibiting orthorhombic symmetry

Orthorhombic arrangement of Tb, Co, and P atoms in TbCo3P2

Tetragonal crystal system observed in Ho5SbAu2 compound

Ba4P3HF24 exhibiting triclinic symmetry

Orthorhombic crystal lattice of KBa2Cd2Sb3 compound

Supplementary Figure S3. Examples of general text type for textual description. A text prompt to generate general text types of textual description and examples of generated general text.

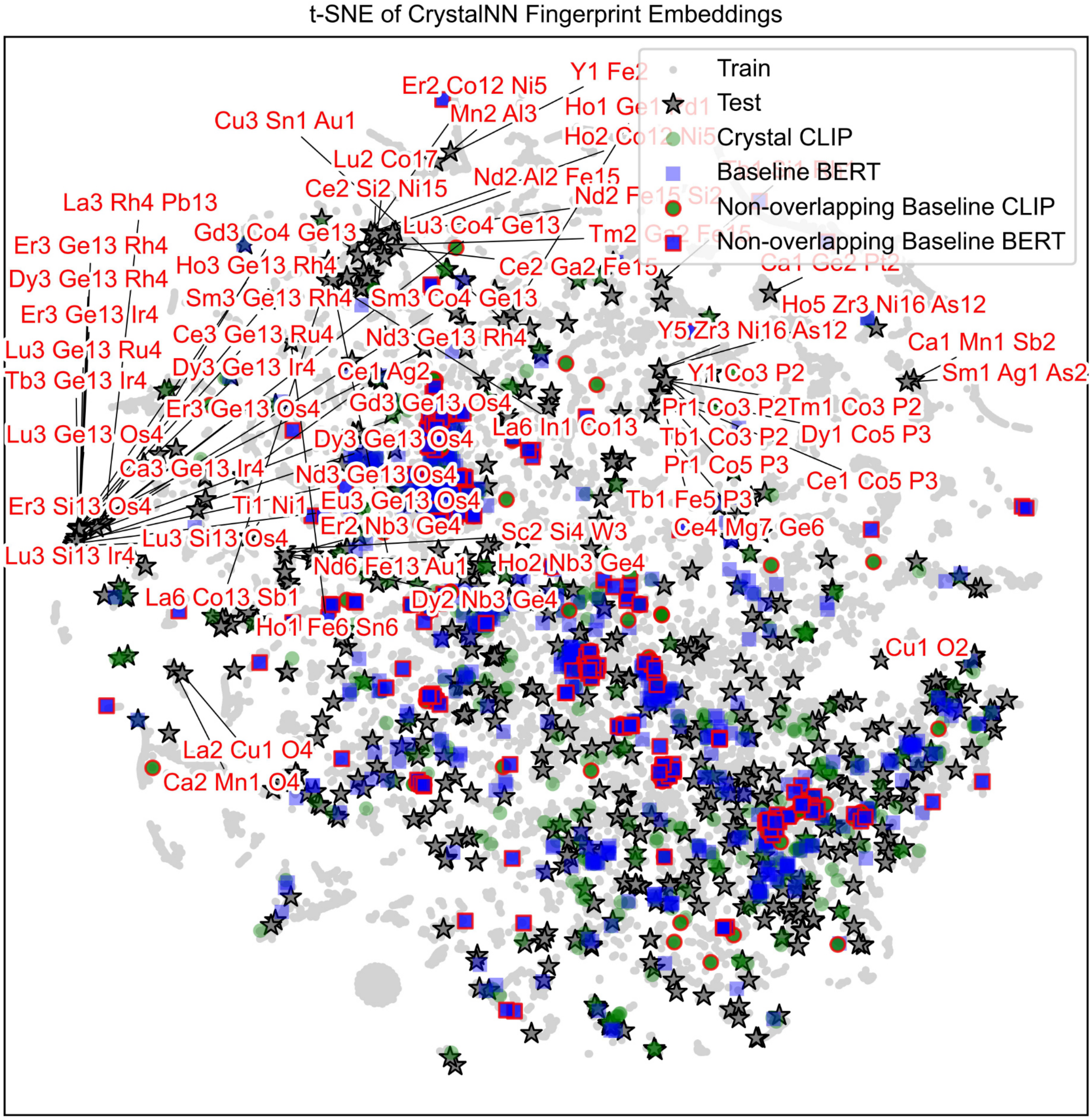

Supplementary Figure S4. A t-SNE plot of structural embedding for train, test, sampled structures. A t-SNE plot of structural embeddings created by Crystal NN Fingerprint for train, test, generated structures by Baseline BERT, Crystal CLIP model when the textual description is general text form. The generated structures that deviate from the test set are highlighted with red edges (non-overlapping), indicating a notable difference from the test set.

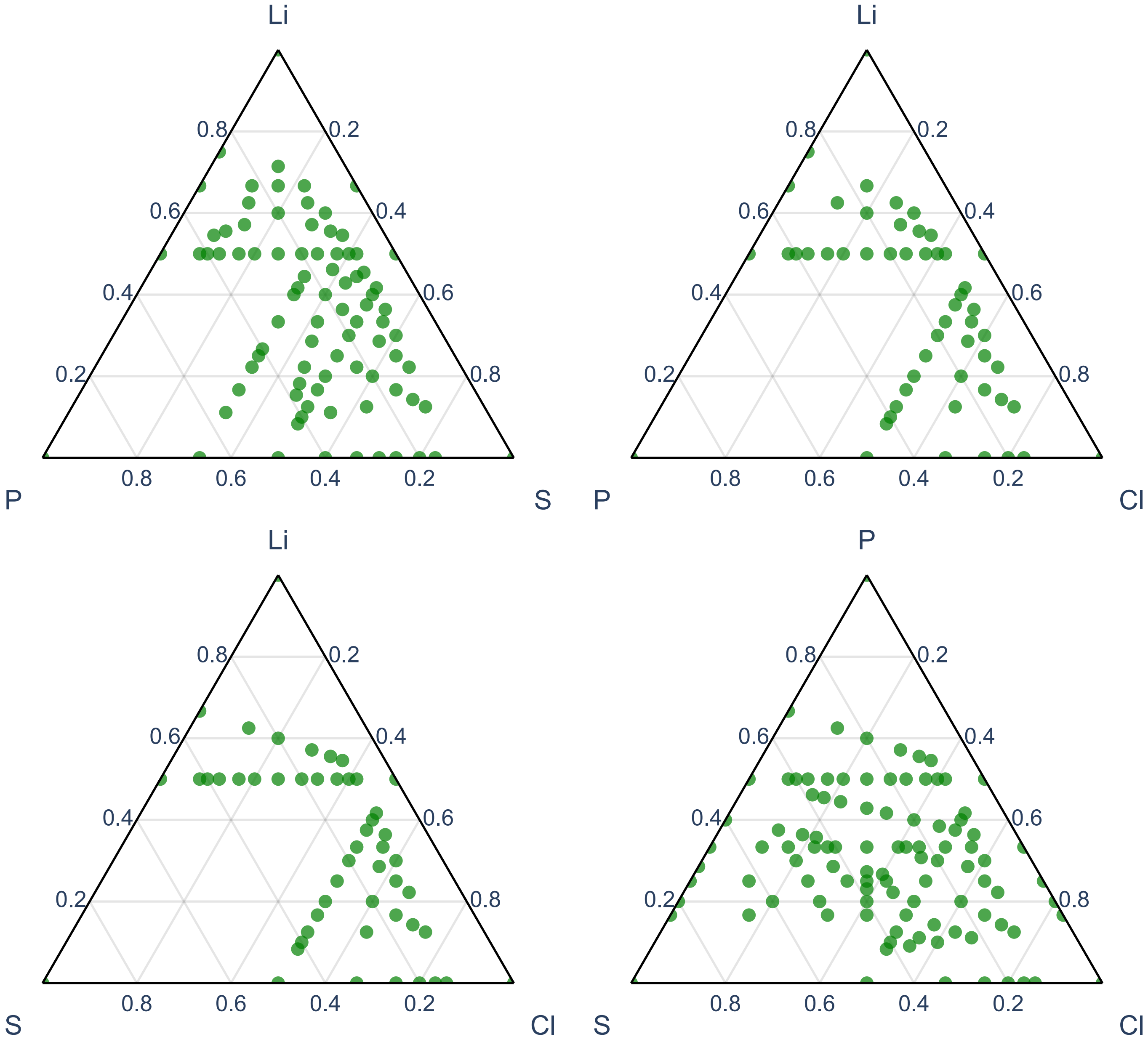

Supplementary Figure S5. Ternary phase diagrams of Li-P-S-Cl with chemical filter. Ternary phase diagrams for Li-P-S-Cl with compositions filtered by the SMACT package, including Li-P-S, Li-P-Cl, Li-S-Cl, P-S-Cl systems.

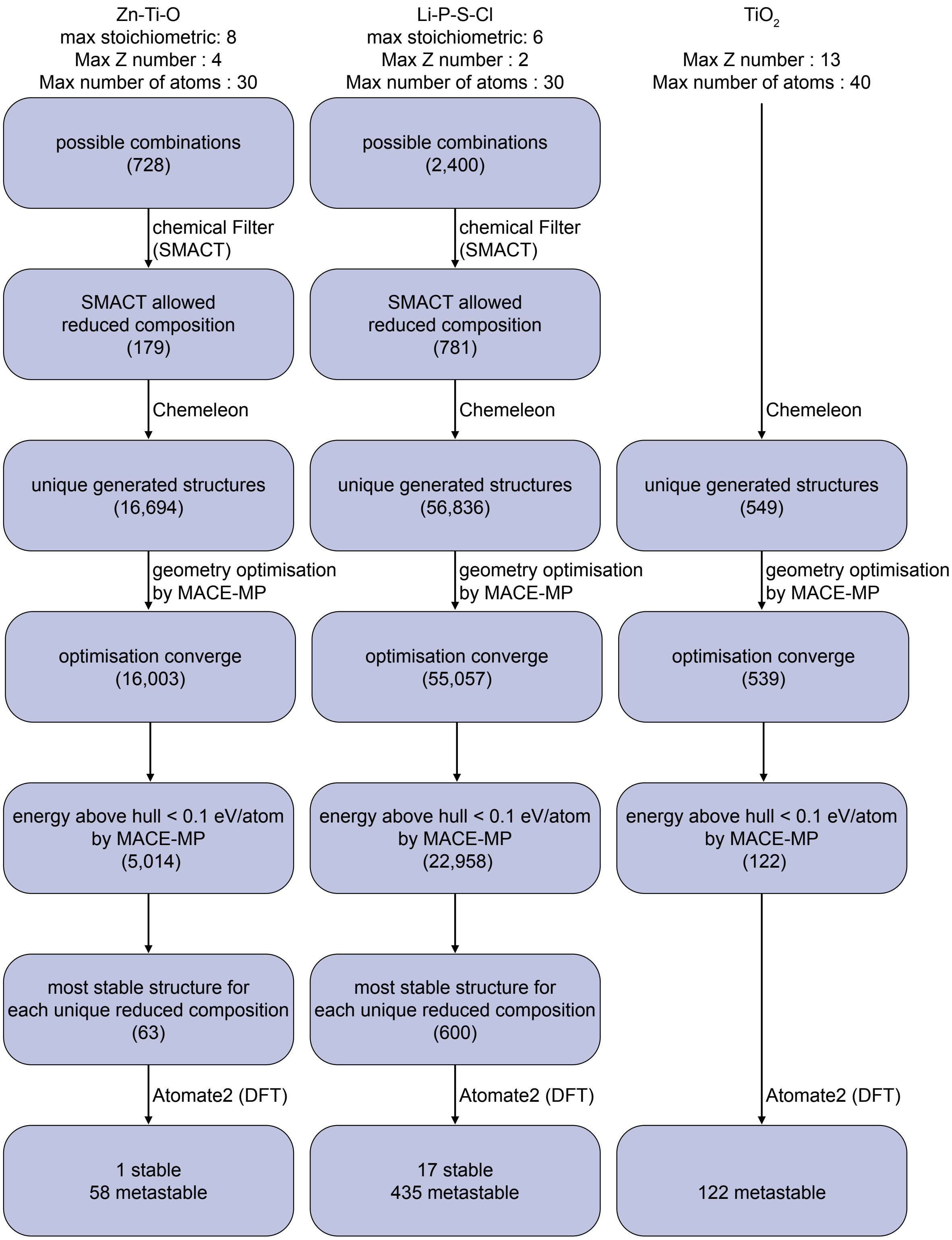

Supplementary Figure S6. A summary of workflows for navigating chemical system. A detailed summary of workflows integrating SMACT, Chemeleon, MACE-MP, Atomate2 for ternary Ti-Zn-O system, TiO_2 polymorph, and quaternary Li-P-S-Cl system.

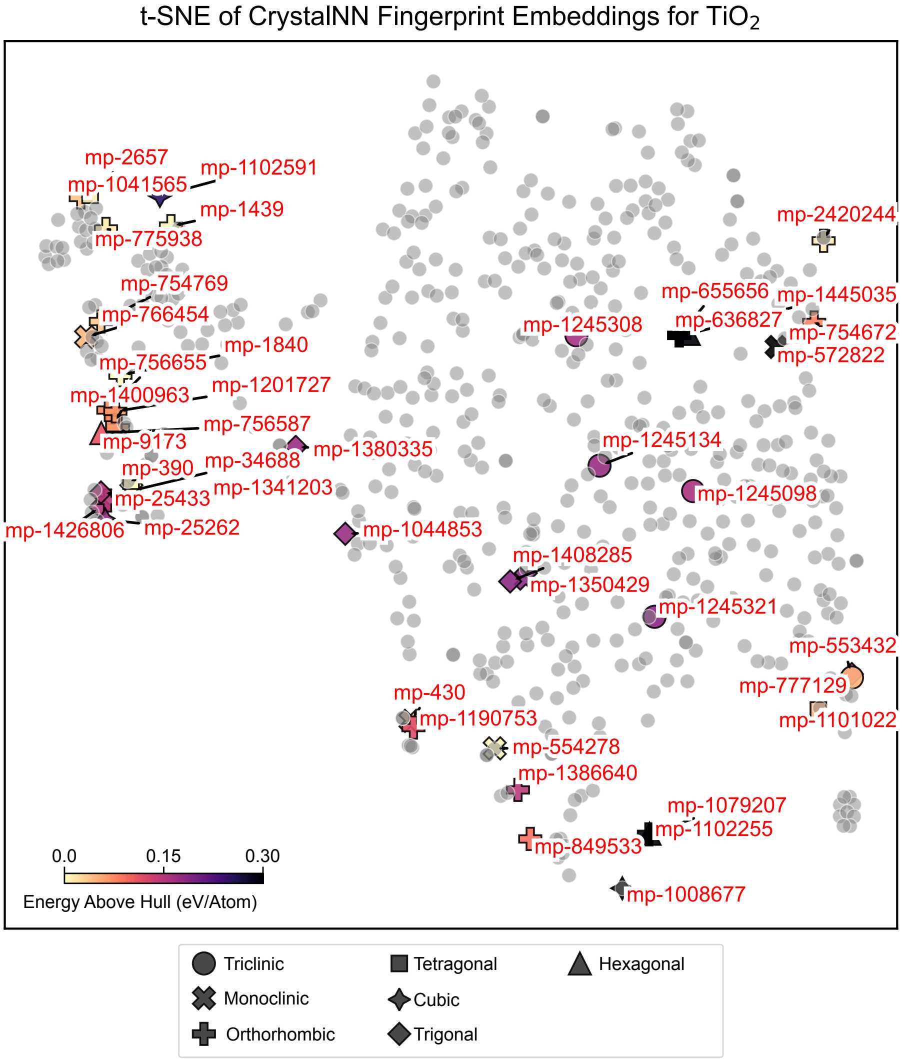

Supplementary Figure S7. A t-SNE plot of structural embeddings for TiO_2 polymorphs system. A t-SNE plot of crystalNN fingerprint embeddings for TiO_2 polymorphs, including both known and generated structures.

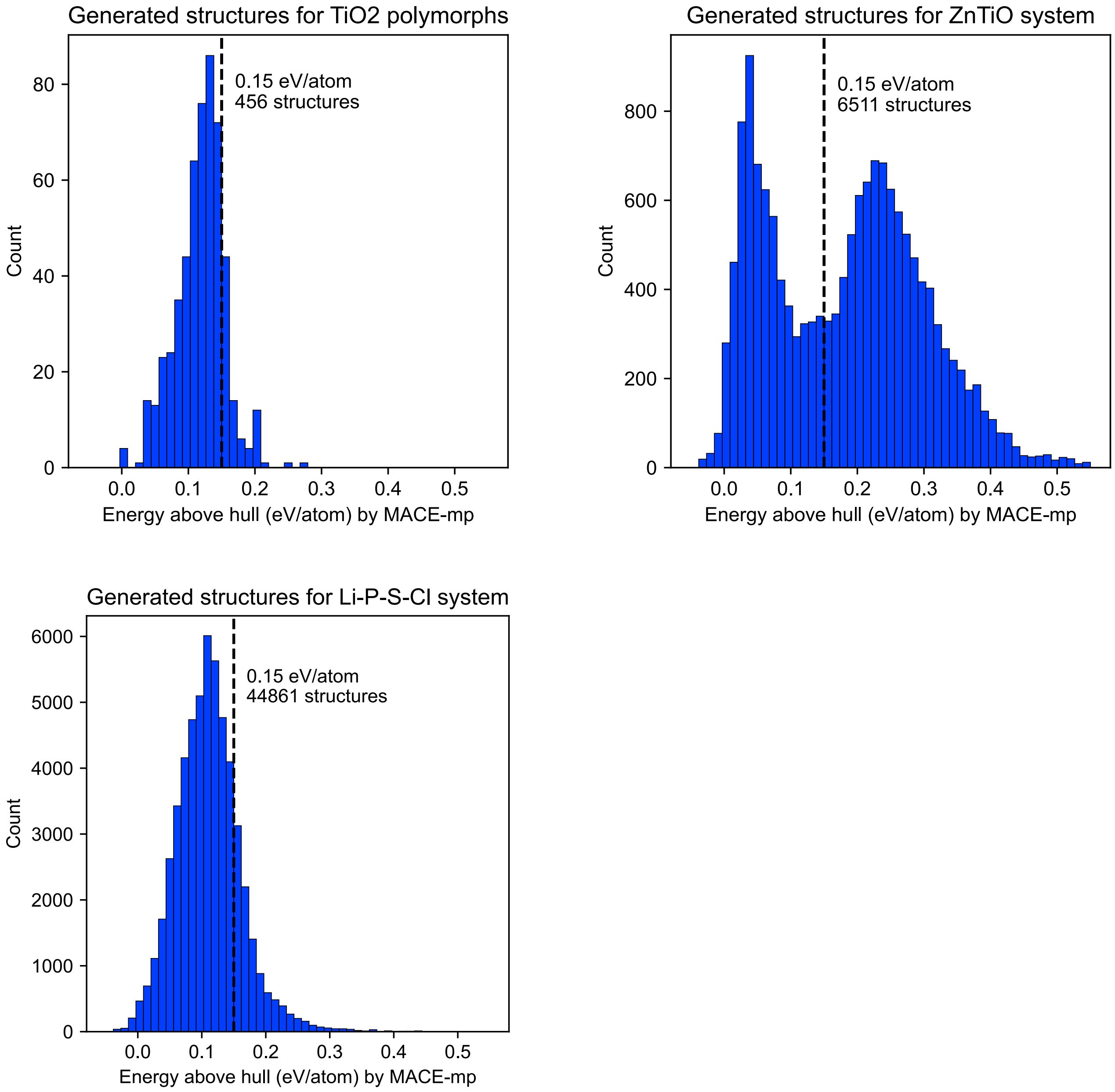

Supplementary Figure S8. Distributions of MACE-MP energies for generated structures by Chemeleon. Distributions of energies obtained from MACE-MP for generated structures by Chemeleon for the binary, ternary and quaternary systems.

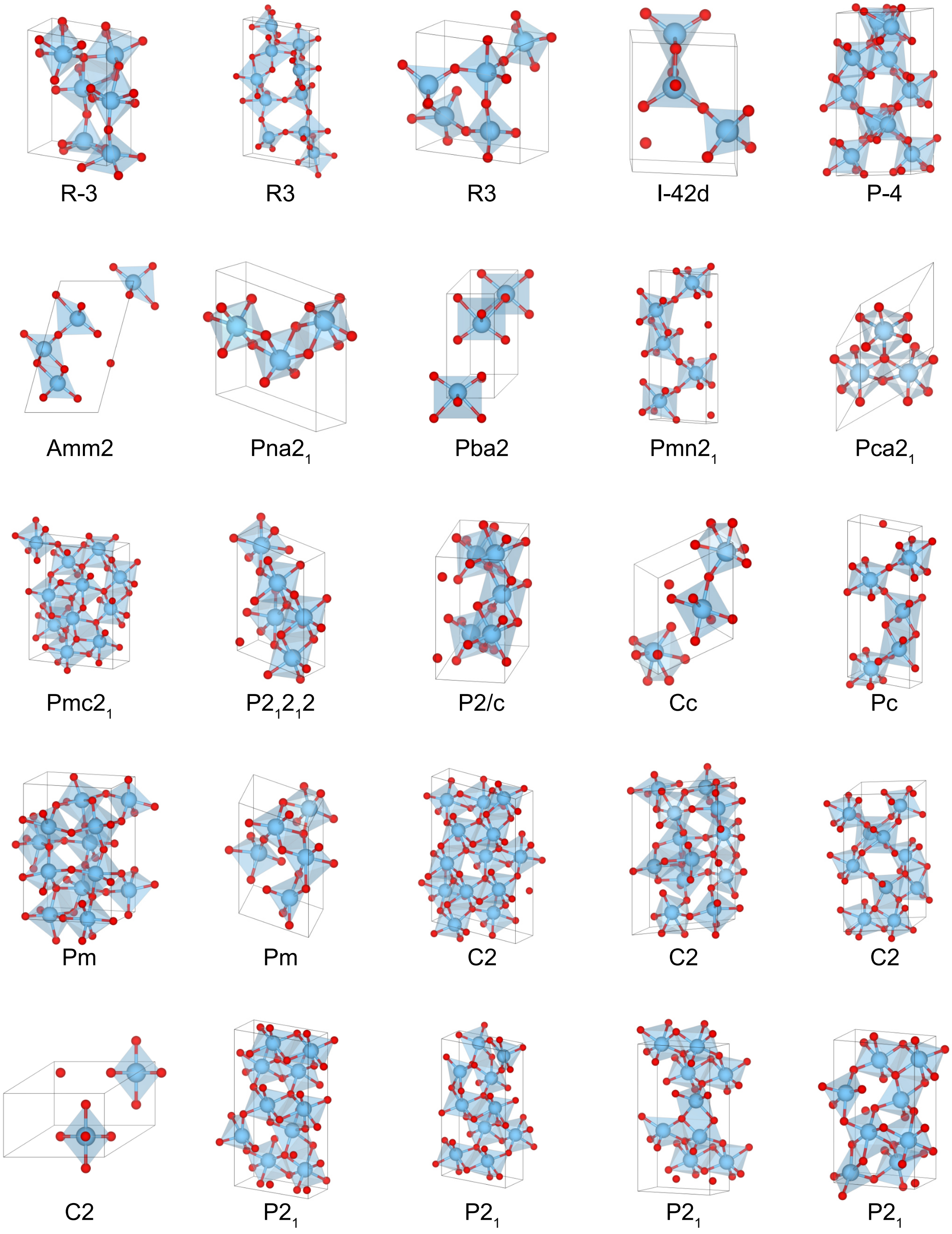

Supplementary Figure S9. Novel metastable TiO_2 polymorphs. The generated metastable TiO_2 polymorphs with previously unobserved space groups.

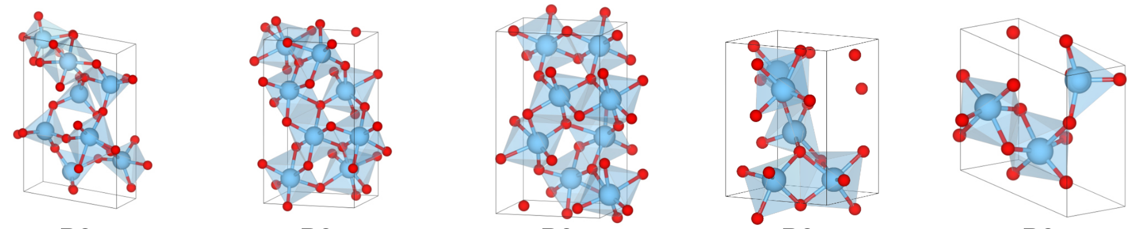

$P2_1$ $P2_1$ $P2_1$ $P2_1$ $P2_1$

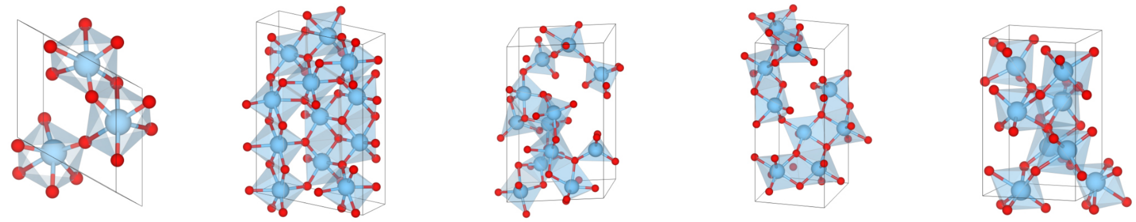

$P2_1$ P2 P-1 P-1 P-1

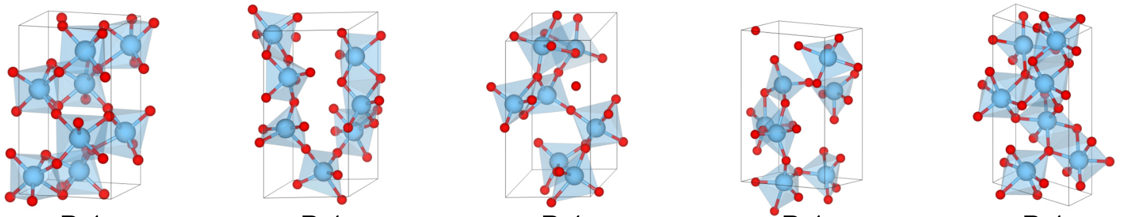

P-1 P-1 P-1 P-1 P-1

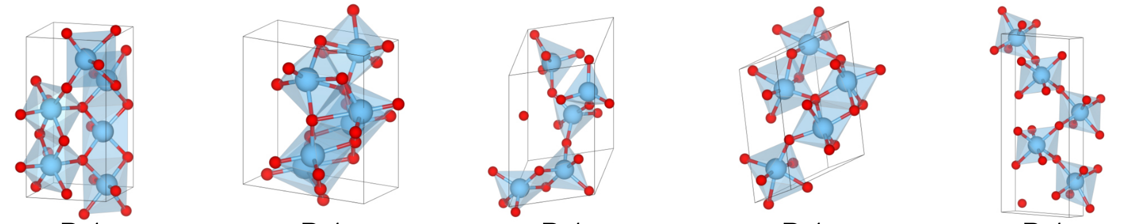

P-1 P-1 P-1 P-1 P-1

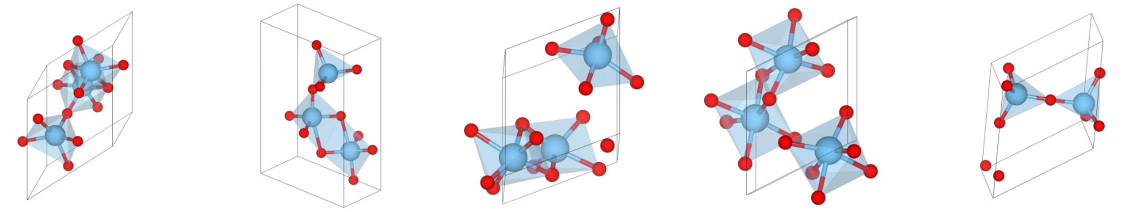

P-1 P-1 P-1 P-1 P-1

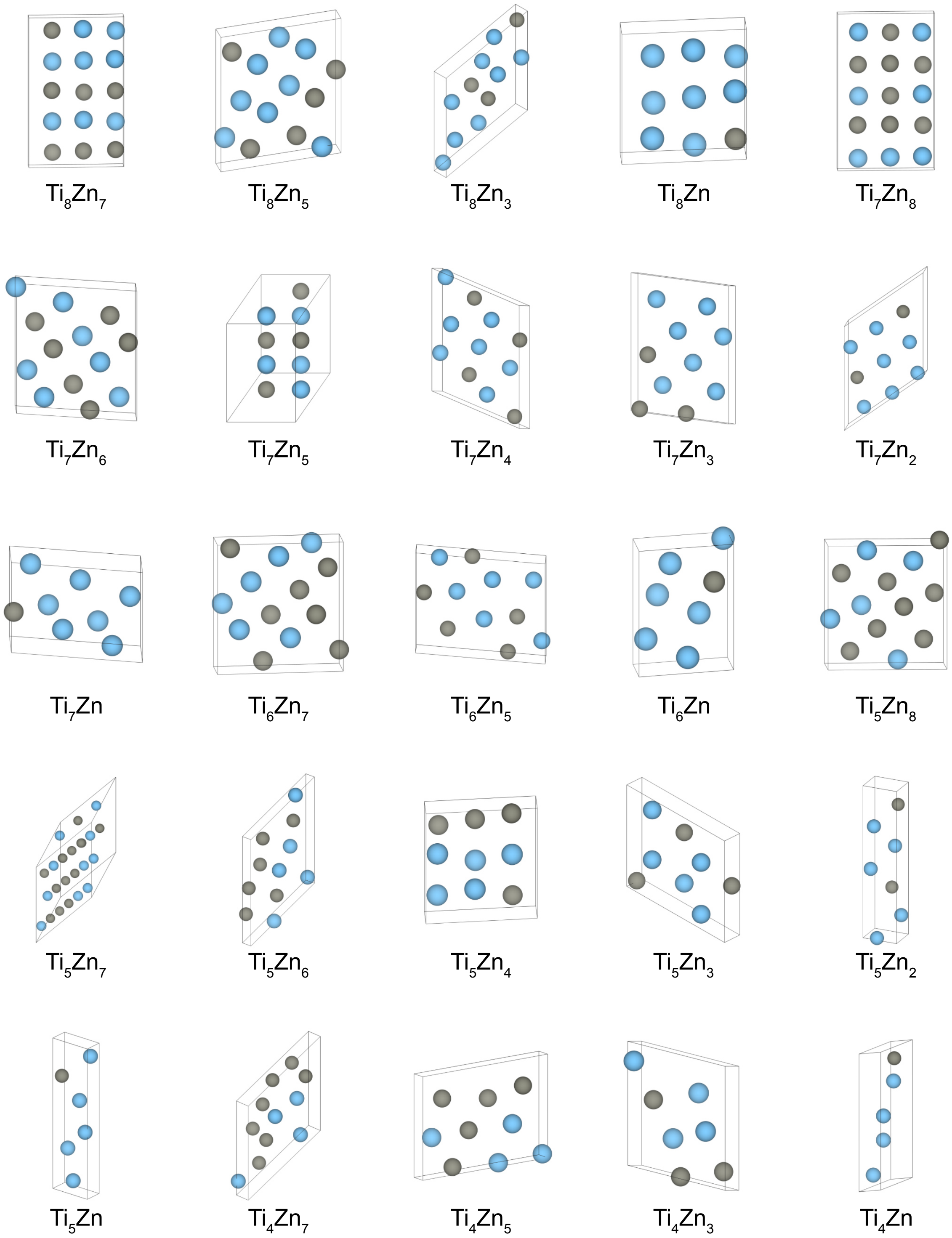

Supplementary Figure S10. Novel generated structures in Ti-Zn-O system. The generated metastable structures for Ti-Zn-O system.

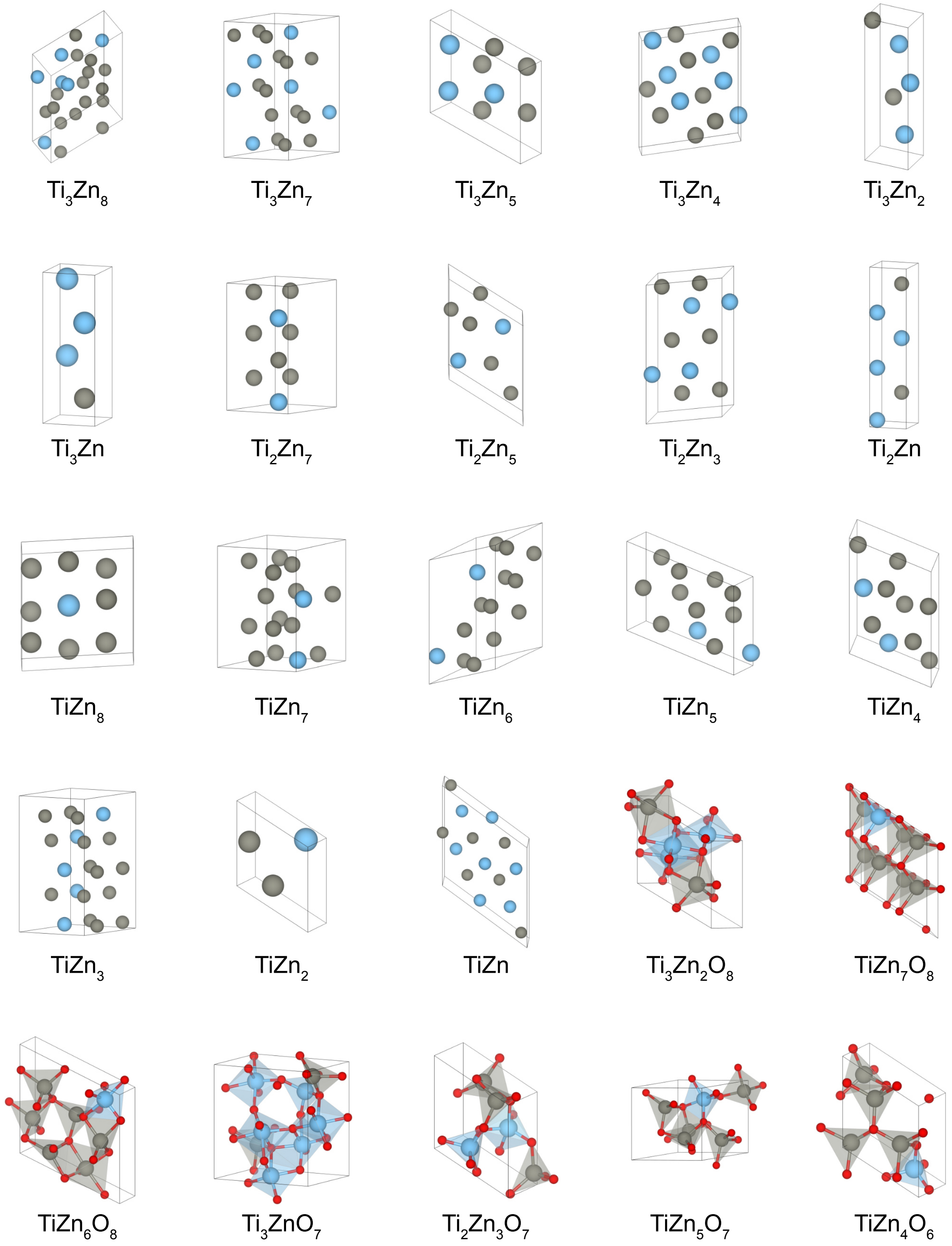
Ti_3Zn_8
Ti_3Zn_7
Ti_3Zn_5
Ti_3Zn_4
Ti_3Zn_2
Ti_3Zn
Ti_2Zn_7
Ti_2Zn_5
Ti_2Zn_3
Ti_2Zn
$TiZn_8$
$TiZn_7$
$TiZn_6$
$TiZn_5$
$TiZn_4$
$TiZn_3$
$TiZn_2$
TiZn
$Ti_3Zn_2O_8$
$TiZn_7O_8$
$TiZn_6O_8$
Ti_3ZnO_7
$Ti_2Zn_3O_7$
$TiZn_5O_7$
$TiZn_4O_6$

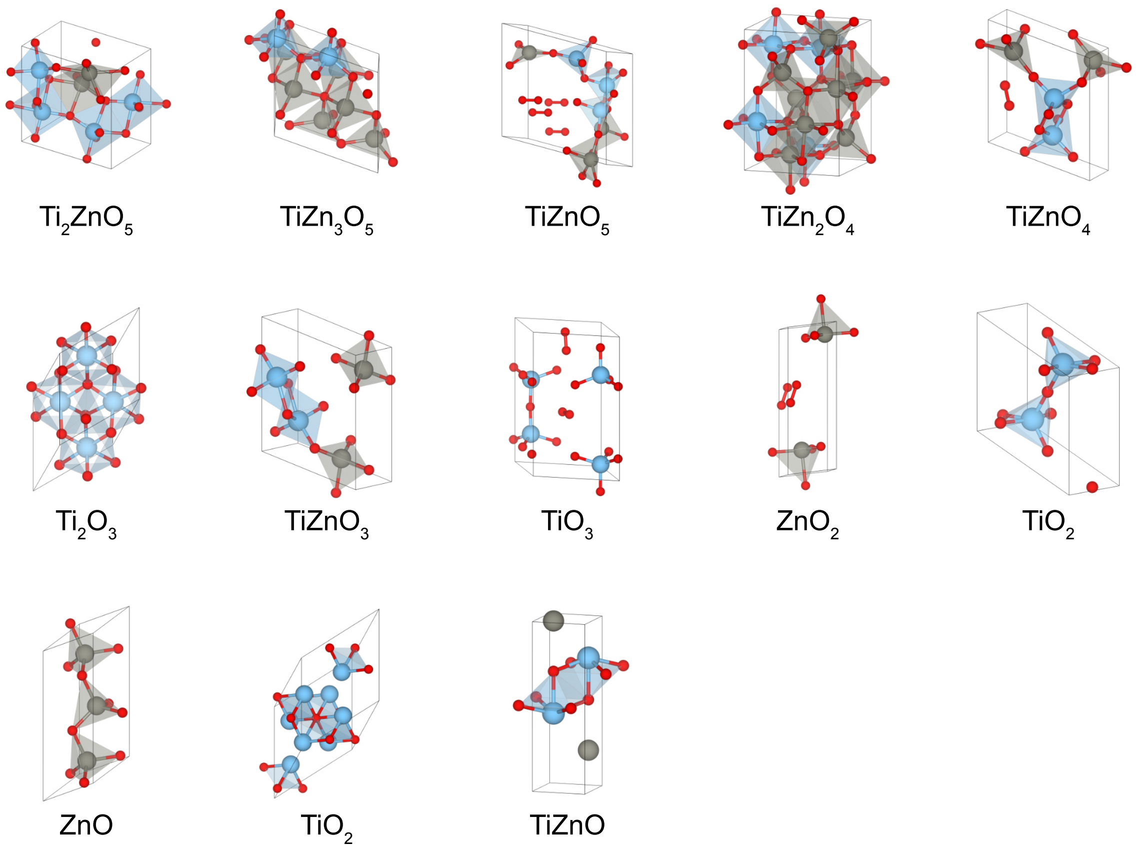
Ti_2ZnO_5
$TiZn_3O_5$
$TiZnO_5$
$TiZn_2O_4$
$TiZnO_4$
Ti_2O_3
$TiZnO_3$
TiO_3
ZnO_2
TiO_2
ZnO
TiO_2
TiZnO

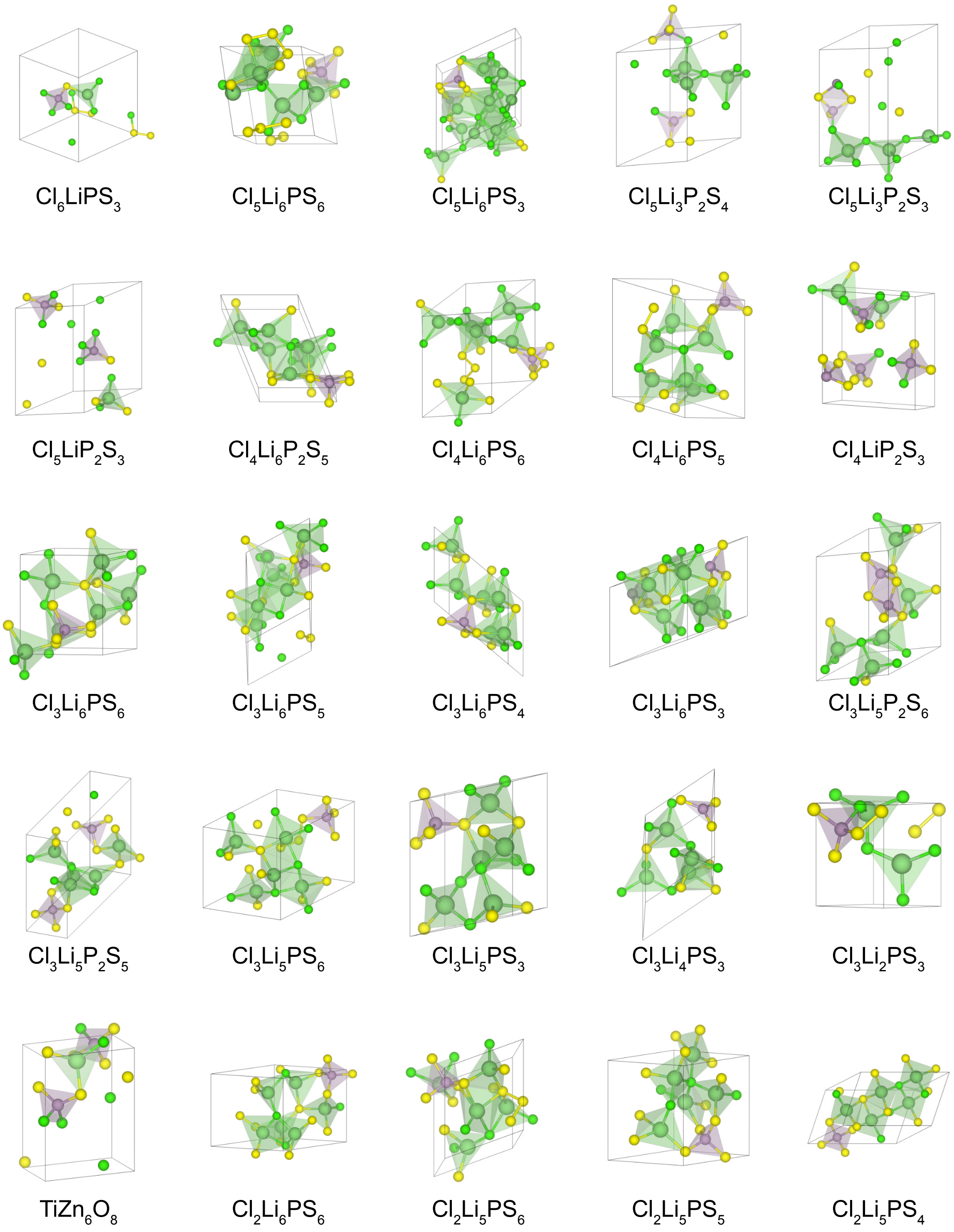

Supplementary Figure S11. Novel generated metastable quaternary structures in Li-P-S-Cl system. The generated metastable structures for Li-P-S-Cl system with 50 lowest energy above the convex hull.

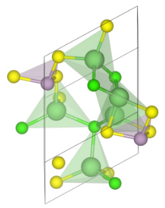

$Cl_2Li_5PS_3$

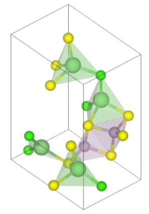

$Cl_2Li_4P_2S_5$

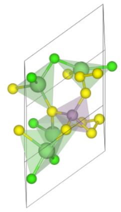

$Cl_2Li_4PS_6$

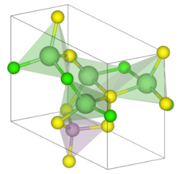

$Cl_2Li_4PS_5$

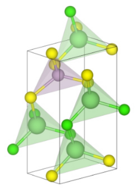

$Cl_2Li_4PS_3$

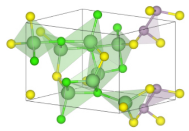

$Cl_2Li_4PS_2$

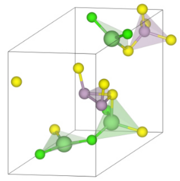

$Cl_2Li_3P_3S_5$

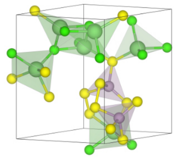

$Cl_2Li_3PS_4$

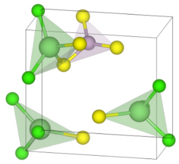

$Cl_2Li_3PS_3$

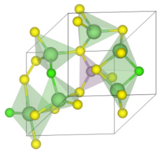

$ClLi_6PS_6$

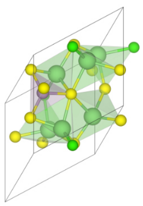

$ClLi_6PS_5$

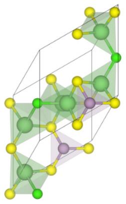

$ClLi_6PS_4$

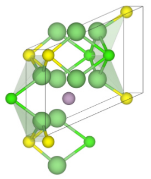

$ClLiPS_6$

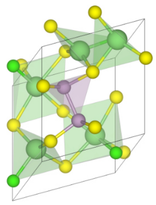

$ClLi_5P_2S_4$

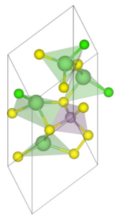

$ClLi_4PS_6$

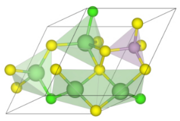

$ClLi_4PS_5$

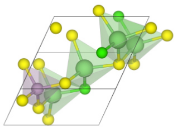

$ClLi_4PS_4$

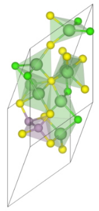

$ClLi_4PS_3$

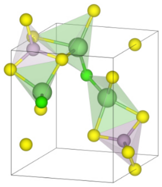

$ClLi_3P_2S_6$

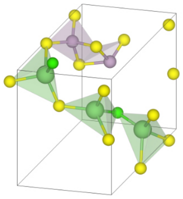

$ClLi_3P_2S_5$

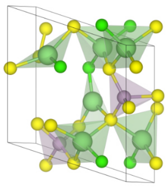

$ClLi_3PS_4$

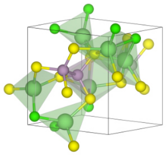

$ClLi_3PS_3$

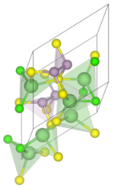

$ClLi_3PS_2$

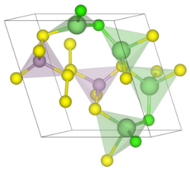

$ClLi_2PS_4$

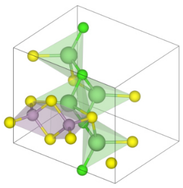

$ClLi_2PS_3$

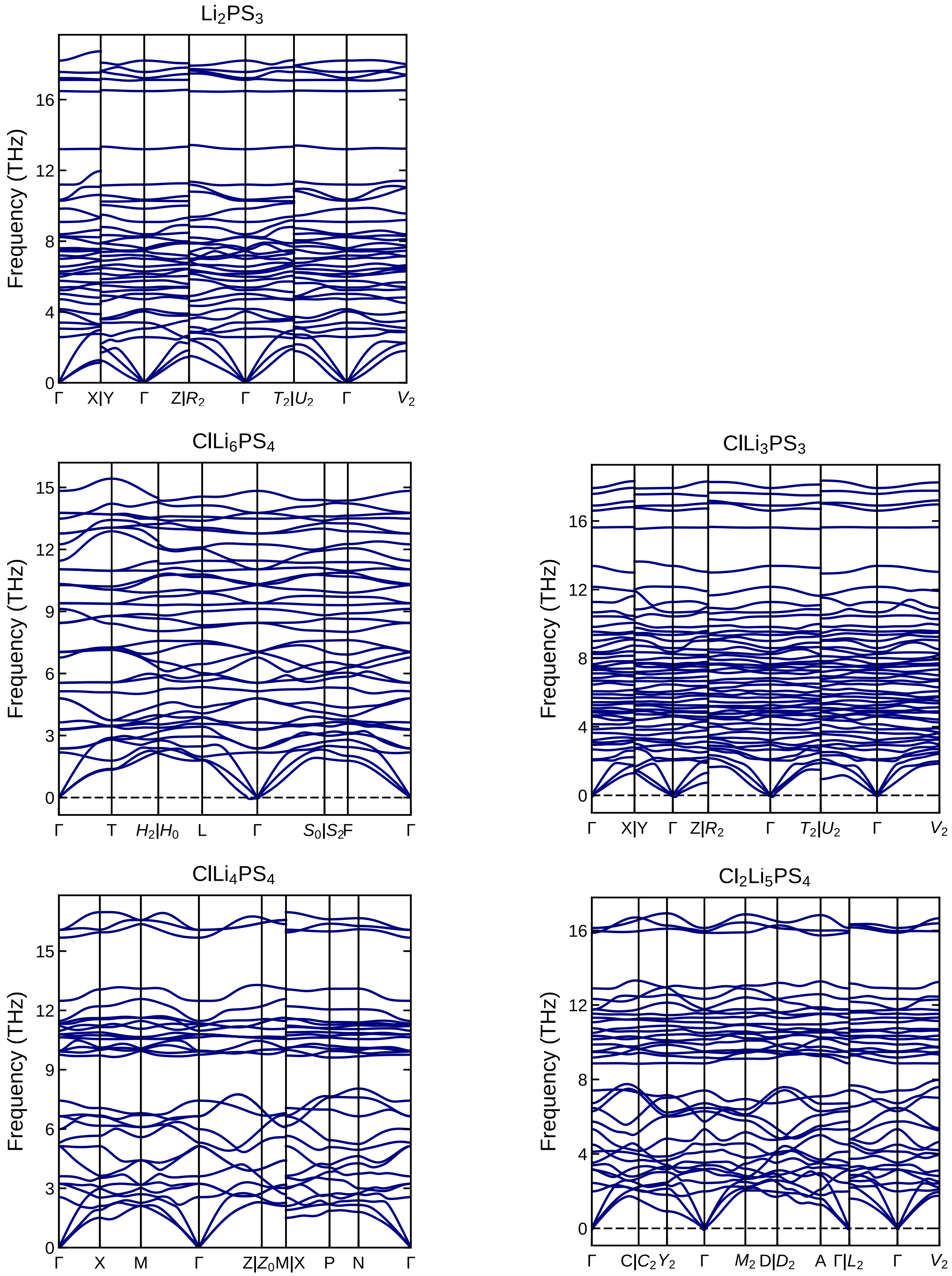

Supplementary Figure S12. DFT phonon band structures in Li-P-S-Cl system. Band structures created by DFT phonon dispersion calculations for stable and metastable structures in Li-P-S-Cl system.

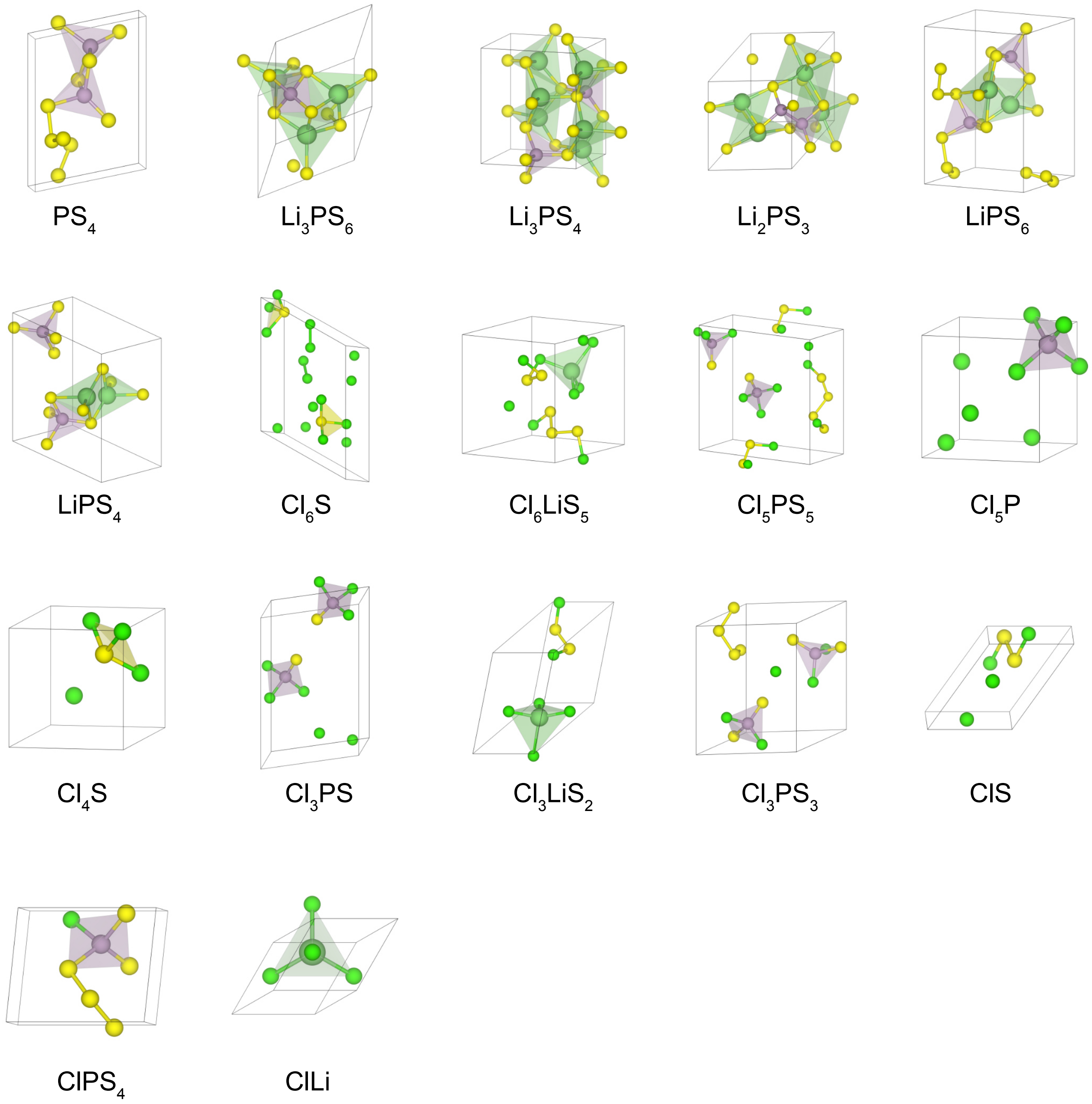

Supplementary Figure S13. Novel generated stable structures in Li-P-S-Cl system. The generated stable structures for Li-P-S-Cl system.

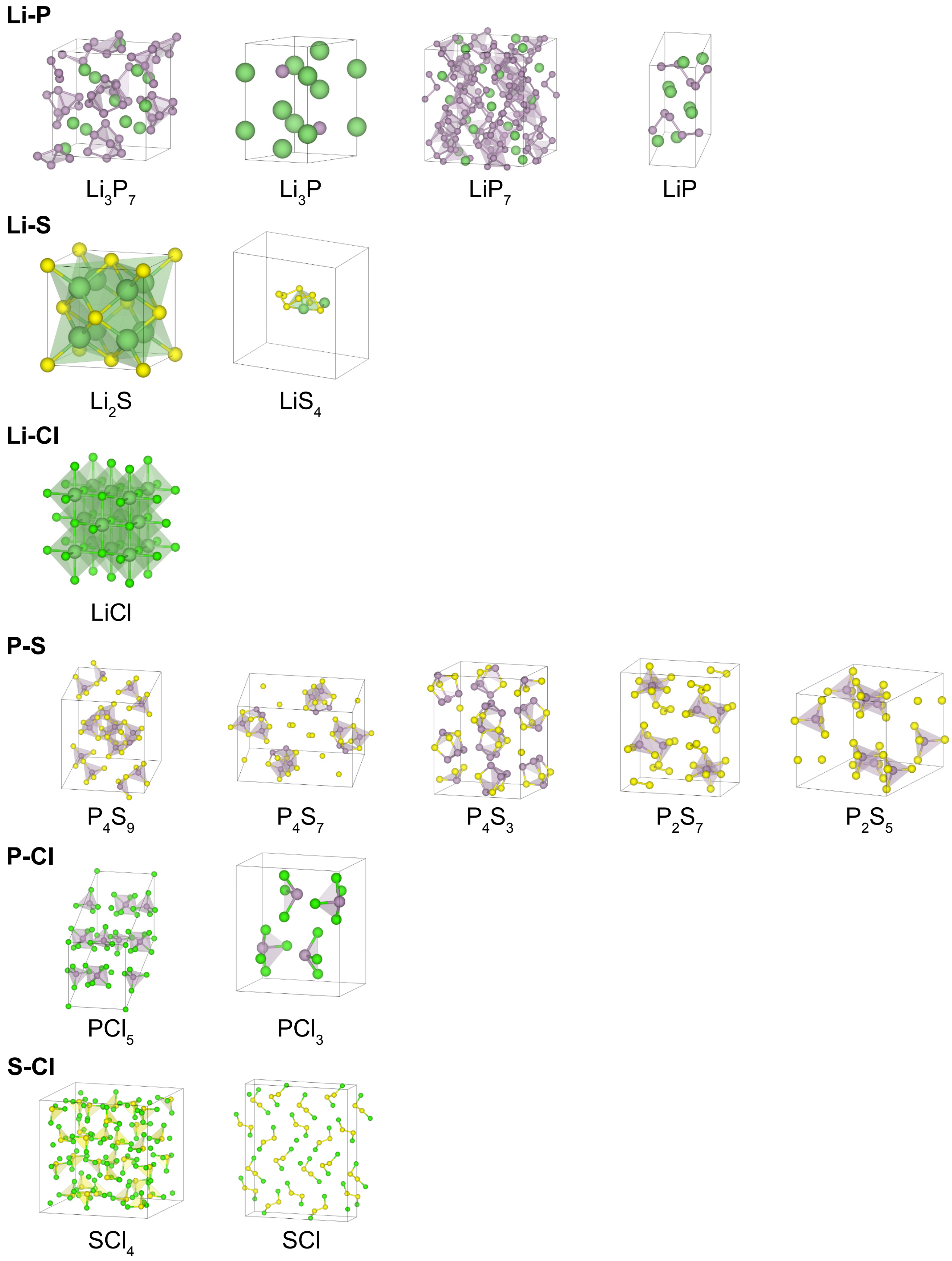

Supplementary Figure S14. Known materials for binary systems in Li-P-S-Cl. Known materials for binary systems in Li-P-S-Cl including Li-P, Li-S, Li-Cl, P-S, P-Cl, S-Cl system.

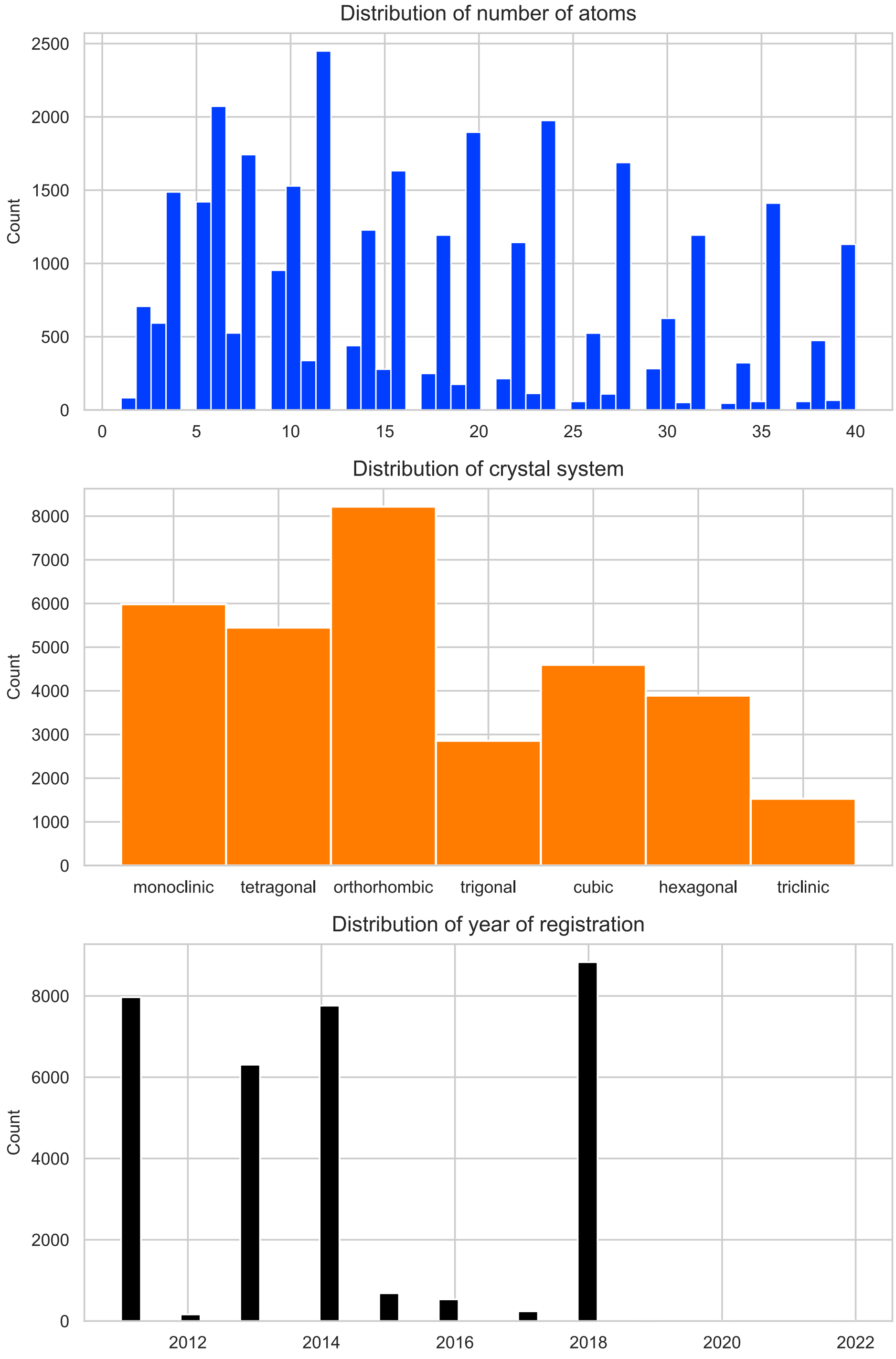

Supplementary Figure S15. Details of MP-40 dataset used for training Chemeleon. Distributions of dataset, including a total of 32,525 structures, for Chemeleon in terms of the number of atoms, crystal system, and registration year of each entry.

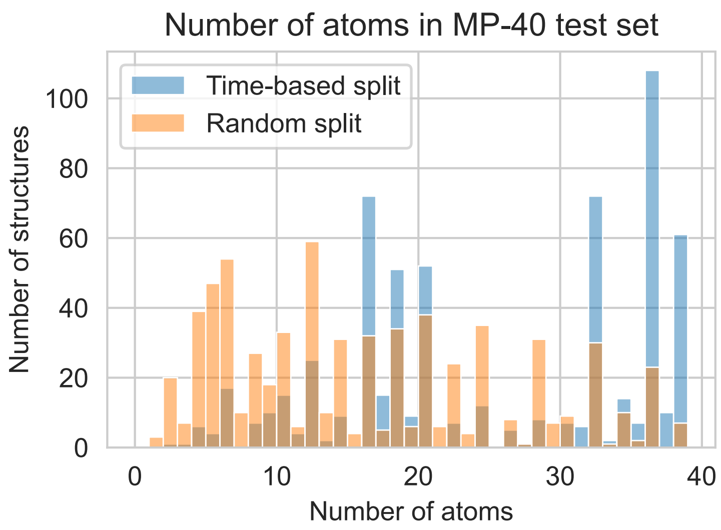

Supplementary Figure S16. Number of atoms in MP-40 test set with different splits. Distributions of number of atoms in MP-40 test set, including a total of 708 structures, with different splits: time-based split (blue) and random split (orange).

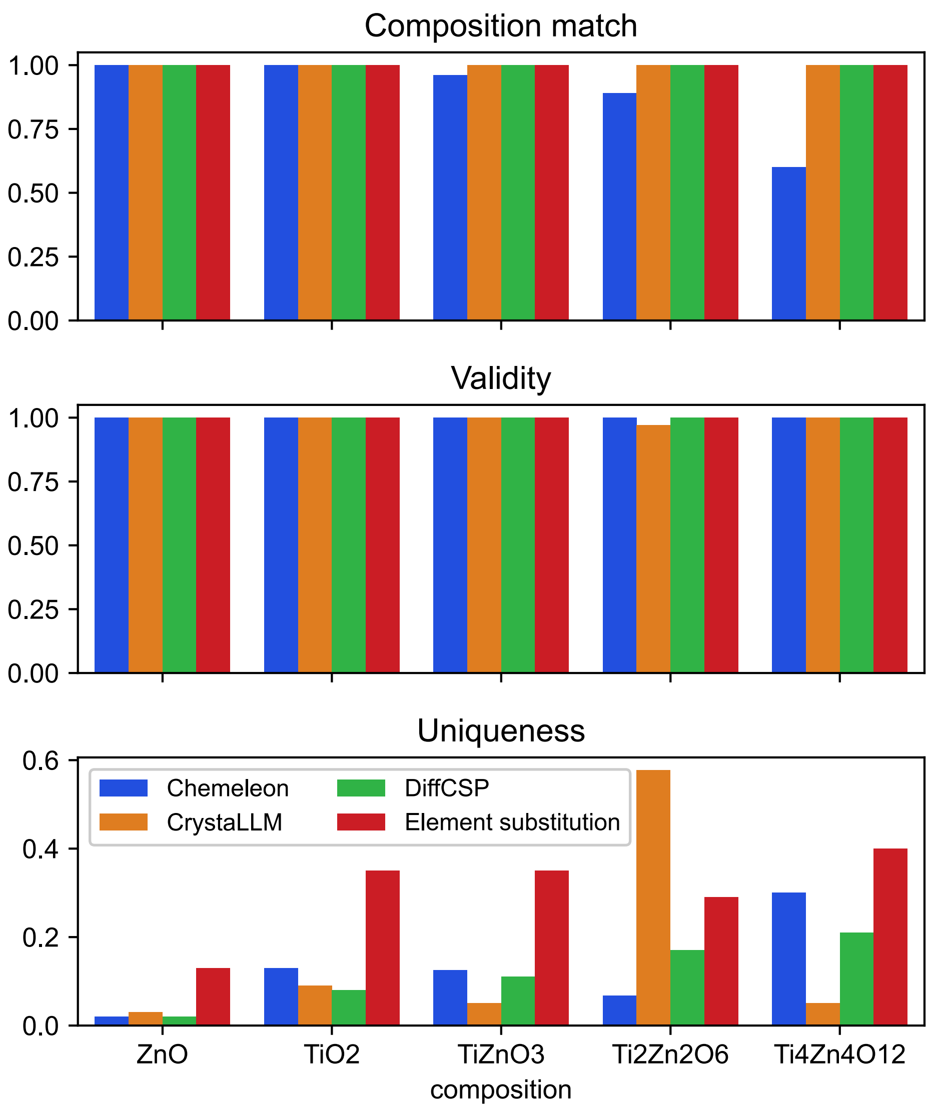

Supplementary Figure S17. Benchmarking Chemeleon with other methods. The composition matching, validity, uniqueness, when sampling 100 structure for ZnO, TiO2, $TiZnO_3$, considering different integer variations of $TiZnO_3$ such as $Ti_2Zn_2O_6$ and $Ti_4Zn_4O_{12}$. Three other methods are assessed including DiffCSP, a diffusion-based crystal structure prediction (CSP) model; CrystaLLM, a large language model (LLM)-based approach; and an element substitution method.

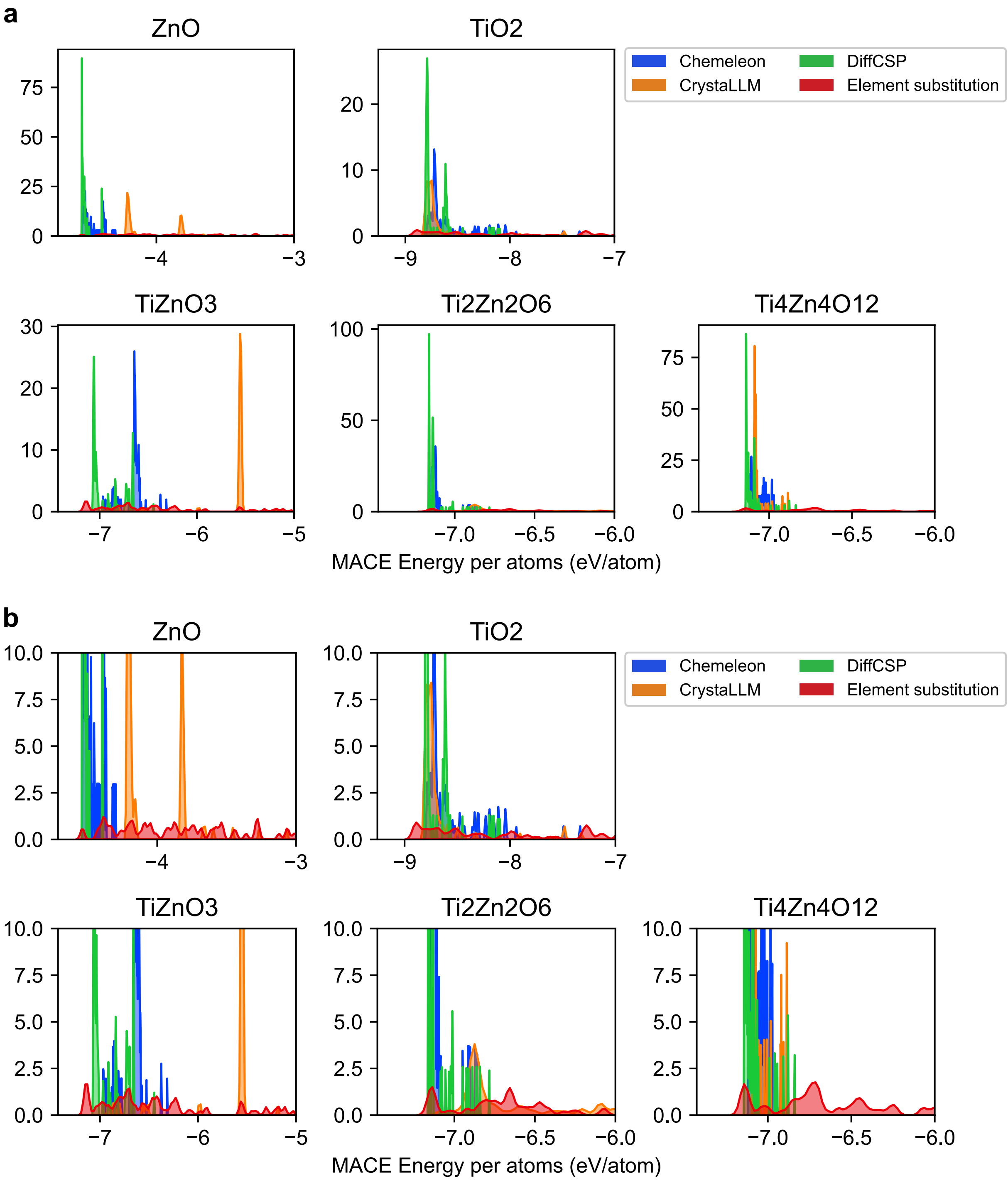

Supplementary Figure S18. Energy distribution in generated crystal structures in benchmarking results. (a) Energy per atom distribution of sampled structures obtained from the MACE-MP-0 model when sampling 100 structure for ZnO, TiO_2, $TiZnO_3$, considering different integer variations of $TiZnO_3$ such as $Ti_2Zn_2O_6$ and $Ti_4Zn_4O_{12}$. Three other methods are assessed including DiffCSP, a diffusion-based crystal structure prediction (CSP) model; CrystaLLM, a large language model (LLM)-based approach; and an element substitution method (b) Same data as in (a) but with a reduced y-axis range for improved visualization.

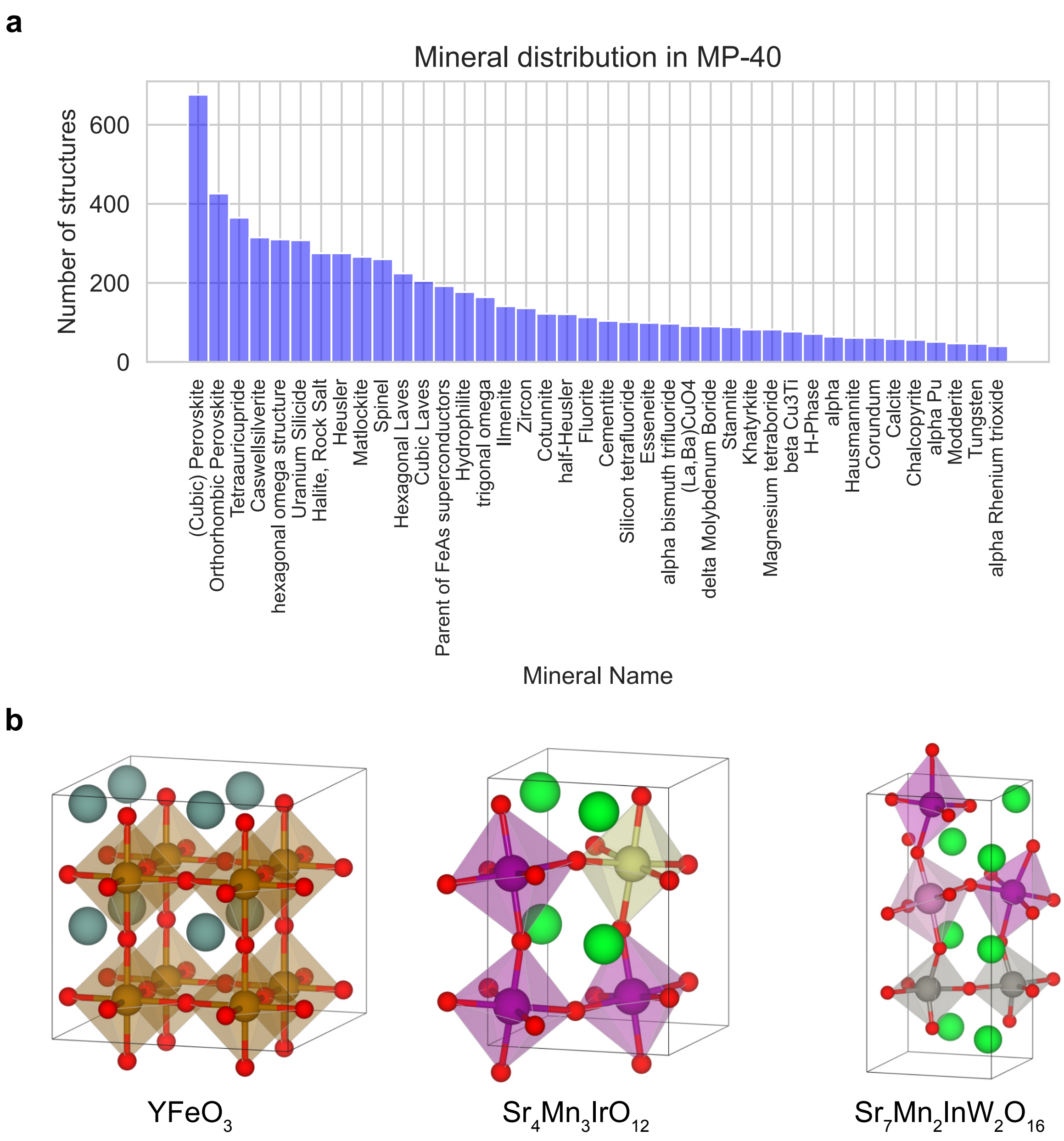

Supplementary Figure S19. Chemeleon trained with mineral names. (a) Mineral name distributions were obtained using RoboCrystallographer with the AFLOW prototype database on a training set of 6,000 entries. (b) Three generated Perovskite structures, which are not overlapped in training and test set. The samples are generated using Chemeleon trained with mineral names.

Supplementary Table S1. Model evaluation on MP-20 dataset. Comparison of crystal structures generated by Chemeleon, DiffCSP, and FlowMM on the MP-20 dataset when sampling 20 structures. The composition match rate and structure match rate indicate the proportion of sampled structures that match the ground truth compositions and structures in the test set. RMSE represents the root mean square error between the sampled structures and the ground truth structures, as implemented in the DiffCSP paper.

Model type	# of samples	Composition Match Rate	Structure Match Rate	RMSE
FlowMM	20	-	76.55	0.0834
DiffCSP	20	-	77.93	0.0492
Chemeleon	20	67.52	36.14	0.0804

Supplementary Table S2. Evaluation on text-guided metrics. Evaluation of the crystal structures generated by Chemeleon in terms of composition matching and crystal system (lattice) matching.

Model type	Textual description	Text encoder	Composition matching	Crystal system matching
Unguided	-	-	0.0 (0.0)	0.20 (0.22)
composition	"Li1 Mn1 O4"	Baseline BERT	0.18 (0.35)	0.51 (0.52)
		Crystal CLIP	0.30 (0.52)	0.60 (0.57)
formatted text	"composition: Li Mn O4, crystal system: orthorhombic"	Baseline BERT	0.11 (0.2)	0.70 (0.69)
		Crystal CLIP	0.27 (0.48)	0.73 (0.72)
general text	"crystal structure of LiMnO4 with orthorhombic symmetry"	Baseline BERT	0.08 (0.14)	0.64 (0.64)
		Crystal CLIP	0.30 (0.53)	0.75 (0.74)

(): Values in parentheses represent scores for structures having fewer than 20 atoms

Supplementary Table S3. Model evaluation on structural metrics with random split. Evaluation of the crystal structures generated by Chemeleon in terms of validity (structural parameters), uniqueness (between generated structures), structure matching (ground truth present), and metastability (energy threshold) composition matching and crystal system (lattice) matching across a batch of 20 samples.

Model type	Text encoder	Validity	Uniqueness	Structure matching	Metastability	Composition matching	Crystal system matching
composition	Crystal CLIP	0.99	0.72	0.41	0.37	0.50 (0.66)	0.61 (0.65)
general text	Crystal CLIP	0.99	0.72	0.43	0.36	0.50 (0.66)	0.84 (0.86)

(): Values in parentheses represent scores for structures having fewer than 20 atoms
