# Supplementary material for: Exploration of crystal chemical space using text-guided generative artificial intelligence
Source: Nat Commun. 2025 May 12;16:4379. doi: 10.1038/s41467-025-59636-y (PMC12069578; doi:10.1038/s41467-025-59636-y)
Supplement: Supplementary file 2 — Description of Additional Supplementary Files [file 41467_2025_59636_MOESM2_ESM.docx]

**Description of Additional Supplementary Files**

Supplementary Data 1: Raw data of performance evaluation values used in Figure 3(a) and (b), as well as the two-dimensional t-SNE embedding vectors of the CrystalNN fingerprint for TiO₂ presented in Figure 4(d).
